# Scaling down annotation needs: The capacity of self-supervised learning on diatom classification

**DOI:** 10.1016/j.isci.2025.112236

**Published:** 2025-03-20

**Authors:** Mingkun Tan, Daniel Langenkämper, Michael Kloster, Tim W. Nattkemper

**Affiliations:** 1 Biodata Mining Group, Faculty of Technology, University of Bielefeld, 33501 Bielefeld, NRW, Germany; 2 Phycology Group, Faculty of Biology, University of Duisburg-Essen, 45141 Essen, NRW, Germany

**Keywords:** Aquatic science, Biological classification, Aquatic biology

## Abstract

In the field of life sciences, diatoms are essential biomarkers for assessing environmental health. Recent advancements in deep learning have transformed the traditionally laborious process of diatom classification through light microscopy. However, commonly used supervised learning methodologies necessitate annotated data, demanding the expertise of seasoned professionals. This study introduces self-supervised learning to tackle the challenge of scarce annotation in diatom classification. First, our results reveal that self-supervised pre-trained models considerably enhance the utilization effectiveness of available annotated data, with benefits increasing as the dataset size decreases. Second, fine-tuning our models with a very small labeled dataset (e.g., 50 samples per class) yields macro-average accuracy comparable to full-supervised levels, thereby reducing the reliance on taxonomic experts by approximately 96.0%. Moreover, extending the pre-training phase to 1600 epochs further reduced the dependency on annotations, achieving comparable accuracy with merely 30 samples per class.

## Highlights

• Our approach reduces labeling to ∼4% (50/class) with full-supervised accuracy
• Our method beats ViT/ResNet across dataset sizes, with larger gains on smaller ones
• Released pre-trained models enhance reproducibility and advance diatom research

## Graphical abstract

**Figure:**
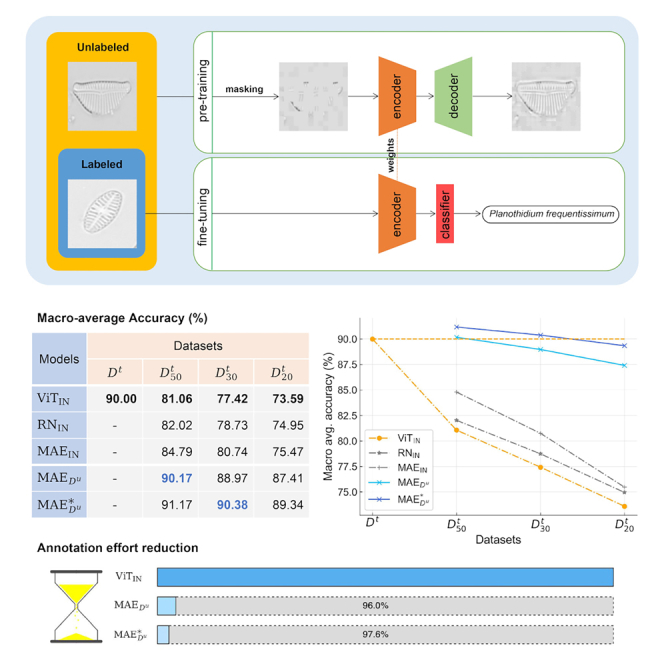

## Introduction

Diatoms play a vital role in life science research as they act as sensitive indicators of water quality and environmental health.^1^^,^^2^ Consistent taxonomic classification is a prerequisite for conducting relevant research.^3^ However, due to their varied morphologies and subtle taxonomic distinctions, diatoms have historically posed identification challenges.^4^^,^^5^ The advent of digital imaging techniques holds the potential to revolutionize diatom taxonomy by transitioning from the time-consuming and labor-intensive process of manual identification using physical light microscope slides to the utilization of digital microscopic slide scans. In recent years, artificial intelligence (AI), particularly deep learning (DL),^6^ has shown significant potential in diatom research by automating the extraction of complex features from raw data such as microscopic images,^7^^,^^8^^,^^9^ thereby supplanting the traditional manual feature extraction methods. These advancements have marked a significant milestone at the intersection of computer vision and diatom classification.

Most previous work on DL-based automatic diatom classification has primarily relied on supervised learning approaches, which necessitate large amounts of annotated data.^9^^,^^10^^,^^11^ Unlike everyday datasets such as ImageNet,^12^ annotating diatom images requires specialized biological knowledge from experienced taxonomic experts, resulting in a scarcity of valuable annotation in this field. Some studies have attempted to address this issue through facilitating the annotation process. Kloster et al.^10^ introduced an integrated digital diatom analysis workflow utilizing the annotation platform BIIGLE 2.0.^13^ Schröder et al.^14^^,^^15^ presented a novel image annotation tool called MorphoCluster. Spaulding et al.^3^ developed an open online platform Diatoms.org. Despite these advancements, obtaining accurate and consistent labeled data remains costly. Additionally, some studies have aimed to optimize the labeling process using active learning techniques.^16^^,^^17^^,^^18^ Although active learning reduces manual labor, it is still time-consuming. These efforts underscore the difficulty, scarcity, and importance of annotation of diatom data.

Therefore, enhancing the utilization effectiveness of available annotated data is imperative due to the high annotation cost. Baharin et al.^19^ demonstrated that improving the quality of low-quality diatom microscopy images can enhance classification performance. However, image pre-processing may lose some important information, especially if over-processed. Others common strategies to maximize the information extracted from given data include applying data augmentation techniques^20^^,^^21^^,^^22^ which need careful design to be effective,^23^ employing model ensemble strategies^24^^,^^25^^,^^26^ but increase computational demands and complicate the training process, and utilizing transfer learning. Widely used convolutional neural network (CNN)-based architectures have been successfully implemented for automatic diatom classification. The performance of different CNNs varies across different datasets, and no single model consistently outperforms others.^9^^,^^11^^,^^27^ Vision transformer (ViT),^28^ which utilizes a self-attention mechanism,^29^ represent another promising feature learning approach, sometimes outperforming CNNs depending on the dataset.^24^^,^^30^ However, these methods do not facilitate learning from unlabeled data.

On the other hand, achieving high performance with minimal annotation is also crucial for reducing costs while ensuring competitive accuracy within diatom classification studies. Especially in biodiversity and life science research, the time experts spend on manual annotation must be spent most efficiently, as data must be annotated by domain experts with sufficient visual expertise and experience. Several methodologies have been explored to address this challenge. For instance, Guo et al.^31^ have introduced a few-shot learning method for marine plankton image classification. This approach synergizes transfer learning with joint training of softmax and center loss functions, aiming to enhance classification accuracy with a limited number of training samples. Nevertheless, few-shot learning may encounter scalability issues when extended to larger or more diverse datasets due to its dependence on a robust base model trained on extensive, representative datasets such as ImageNet. This reliance often limits its adaptability to unconventional datasets, where significant domain shifts can adversely affect model performance. Additionally, some studies have investigated unsupervised learning approaches. Pastore et al.^32^ presented an unsupervised method using Fuzzy k-Means that achieves performance comparable to supervised methods by classifying and detecting anomalies in plankton species with minimal supervision. Furthermore, Pastore et al.^33^ introduced an unsupervised learning pipeline employing pre-trained neural network features and a Variational Autoencoder (VAE) to accurately classify plankton microorganisms from image data. However, traditional clustering-based unsupervised learning methods often struggle with the noise and outliers, challenges that are particularly prevalent in diatom data.^10^

**Figure 1:**
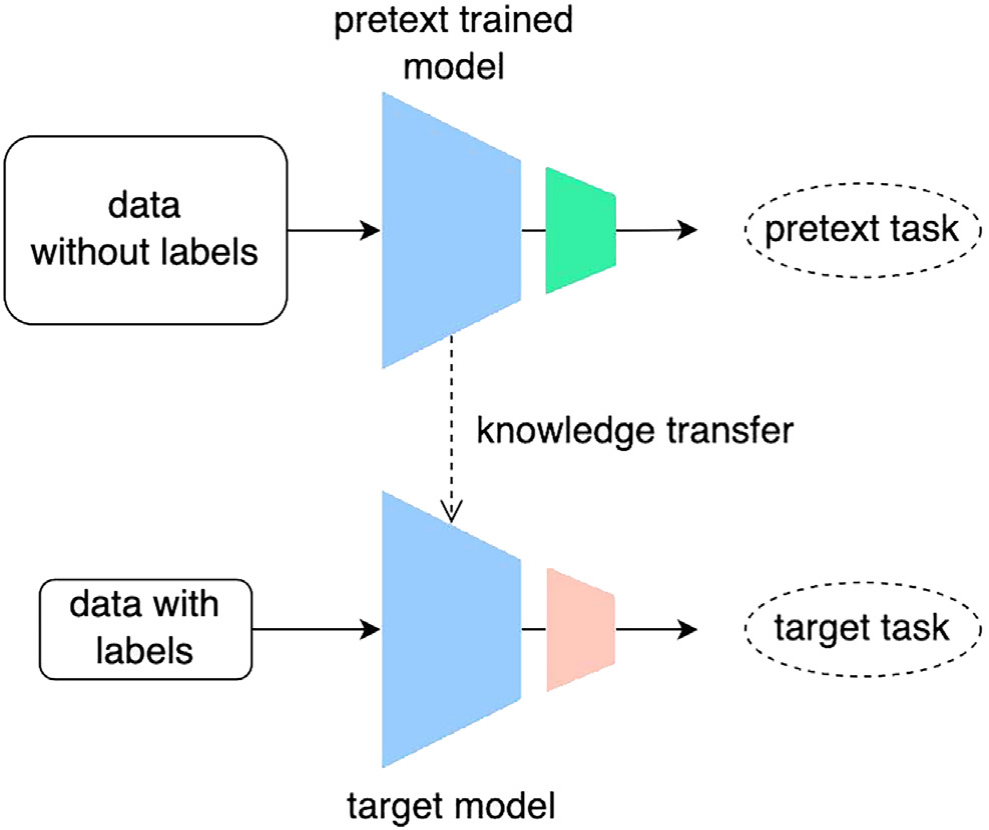
Illustration of self-supervised learning (SSL) workflow At first, the model is pre-trained on a pretext task utilizing unlabeled data, enabling it to capture meaningful features representations inherent in the unlabeled data. Subsequently, the representations are fine-tuned with labeled data to perform a specific downstream task, such as image classification.

Self-supervised learning (SSL), a branch of unsupervised learning, has emerged as a promising approach in image classification, offering a paradigm shift from traditional supervised learning methods. Unlike clustering algorithms that group data into clusters, SSL is designed to extract meaningful feature representations directly from the data. This extraction is facilitated by pretext tasks that derive supervisory information from the data itself, thus enabling the subsequent fine-tuning on these learned representations in downstream tasks like classification with minimal annotated data (Figure 1). SSL can be broadly categorized into two primary types based on their pretext tasks: joint-embedding and self-prediction techniques. Joint-embedding techniques include contrastive learning methods like SimCLR^34^ and MoCo,^35^ as well as negative-free methods like BYOL^36^ and SimSiam.^37^ Self-prediction methods encompass approaches such as innate relationship prediction methods, as presented by Xu et al.,^38^ and generation methods, like masked autoencoders (MAE).^39^ Recently, Schanz et al.^40^ explored the effectiveness of the contrastive learning-based SimCLR^34^ method in marine life imagery data captured in natural underwater environments, significantly reducing the need for annotated data in marine life detection and classification.

In this work, we introduce an SSL approach to effectively and efficiently enhance the classification of microscopy-based diatom data, employing the MAE. Unlike the contrast learning method used by Schanz et al.,^40^ which relies heavily on the quality and diversity of augmentations during pretext task training, we adopt a different method that focuses on reconstructing masked portions of input images. We simulate two distinct scenarios in this work. In the first scenario, termed ‘Comparative MAE Performance’, we employ a fully annotated dataset D to train classifiers under the classical supervised DL paradigm and the MAE. This setup enables us to explore the optimal use of annotated data and assess the impact of the MAE on classification performance. We compare outcomes using 100%, 50%, and 20% of D, respectively. The second scenario, referred to as ‘Annotation Effort Reduction Study’, focuses on achieving high classification performance with significantly smaller datasets, comprising only 50, 30, and 20 annotated samples per class. This scenario investigates the effect of MAE pre-trained with additional unlabeled data from the same domain, which could be sourced from citizen science projects or derived through computational segmentation of whole slide diatom images. To the best of our knowledge, this is the first work to employ SSL for diatom classification. Our study makes three main contributions:

• Our approach consistently outperforms both ViT-L and ResNet-50 methods across various dataset sizes. Notably, the performance advantage increases as the dataset size decreases.
• With only 50 annotated samples per class, our model achieves a macro-average classification accuracy comparable to that of a ViT-L model trained on the full dataset comprising 46294 annotated samples, thereby reducing the annotation effort of domain taxonomic experts by approximately 96.0%. Furthermore, extending the pre-training duration from 800 epochs to 1600 epochs further reduces the demanding on annotated data from 50 to 30 samples per class while maintaining comparable accuracy.
• We will publicly release our two pre-trained models (trained for 800 epochs and for 1600 epochs) to enhance reproducibility and encourage further advancements in the diatom research field. The models are available for download at the following link: Download File.

## Results

### Materials

**Figure 2:**
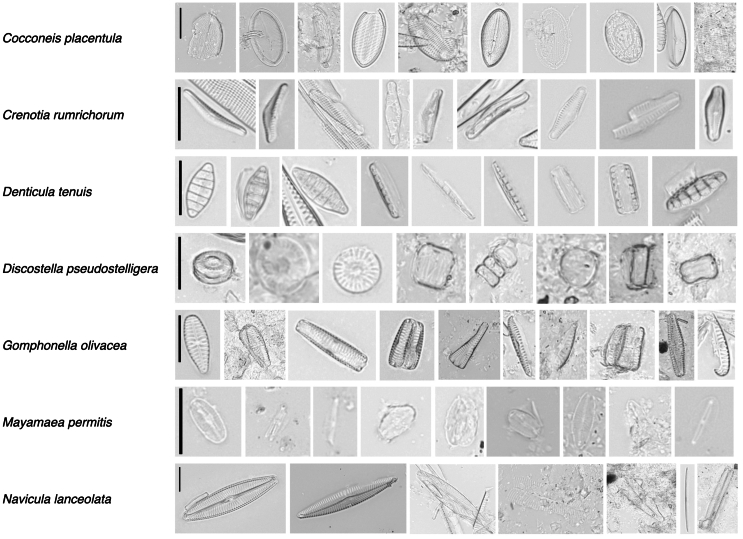
Representative diatom samples with their annotations This figure displays various diatom samples from the dataset used in this study, annotated with their taxonomic names. Each row corresponds to a distinct diatom taxon, showcasing a series of images that highlight the diversity of the morphological characteristics captured within the dataset. The black scale bars, representing a length of 10 μm, are displayed only on the first examples on the left in each row. The other examples in each row have been rescaled for the sake of display aesthetics and compactness. These micrographs exemplify the variability and complexity inherent in the taxonomic classification of diatoms.

**Figure 3:**
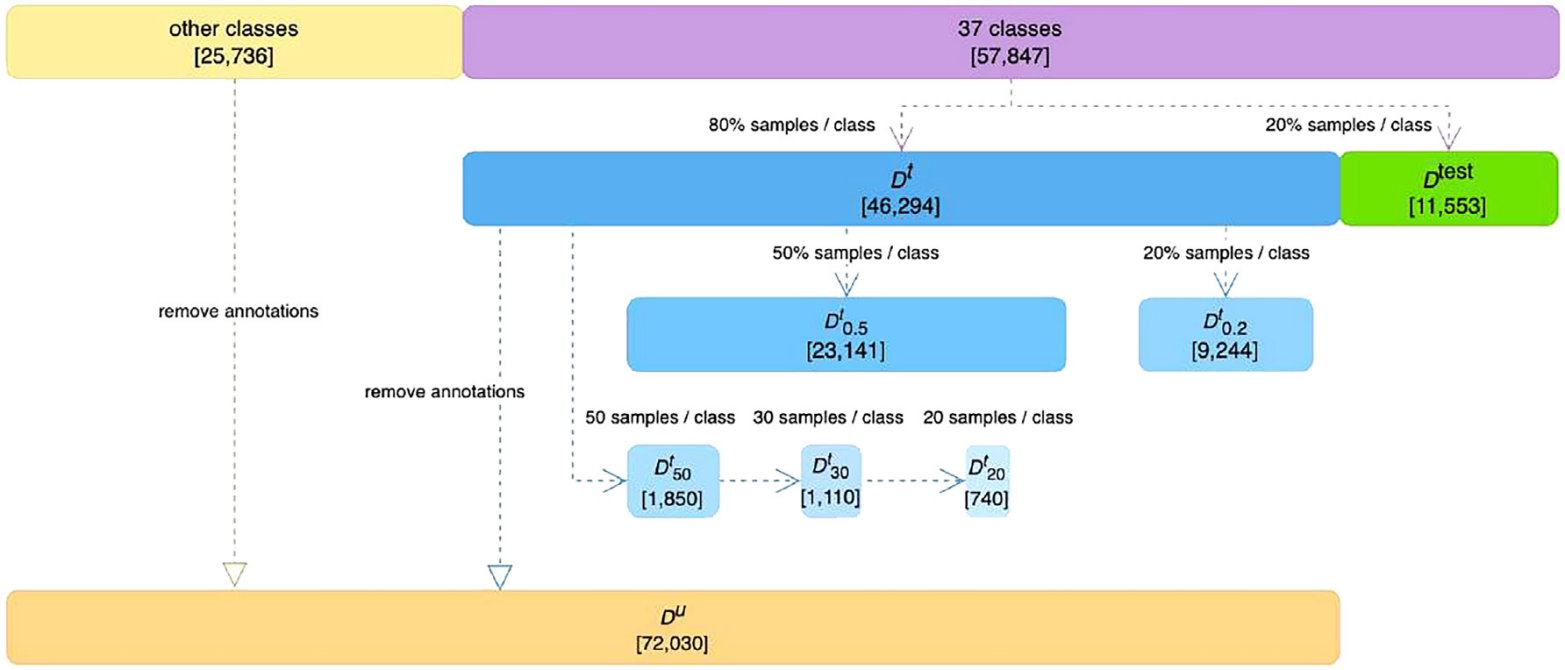
Structure of the datasets utilized in the study This figure visualizes the structure of the datasets used in our experiments. In order to guarantee a sufficient number of images for a reliable test set, we chose all classes with at least 300 images each. This yielded 37 unique classes, totaling 57,847 images. The dataset was split into a primary training set ($D^{t}$, constituting 80% of the data) and a test set ($D^{\text{test}}$, comprising the remaining 20%). To generate an unlabeled dataset, $D^{u}$, annotations were removed from both the training set and any classes containing fewer than 300 samples. To simulate scenarios with varying training data sizes, we created reduced training subsets: $D_{0.5}^{t}$ and $D_{0.2}^{t}$ by randomly sampling 50% and 20% of the data from each class in the training set, respectively, and $D_{50}^{t}$, $D_{30}^{t}$, and $D_{20}^{t}$ by randomly sampling 50, 30, and 20 samples from each class in the training set, respectively. The number of images in each subset is specified in brackets.

The dataset employed in this study constitutes the largest real-world diatom image dataset to date,^41^ consisting of 83,583 images that represent 611 distinct diatom taxa. Figure 2 displays sample diatom images from this dataset, accompanied by their corresponding annotation. Multiple images of a particular diatom taxa are displayed in each row, showcasing the diversity and complexity inherent in the dataset and underscoring the substantial challenge of taxonomic classification that this dataset poses. For our experiment, aiming at ensuring an adequate sample size to form a robust test set, we selected all classes each containing at least 300 images, which resulted in 37 distinct classes, comprising a total of 57,847 images. We then divided them into a primary training set, denoted as $D^{t}$, comprising 80% of the data, and a test set, denoted as $D^{\text{test}}$, making up the remaining 20%. Additionally, we eliminated the classification labels from the training set and from other classes that contained fewer than 300 samples, merging them into an unlabeled dataset called $D^{u}$. To accommodate a range of data availability scenarios, particularly under conditions of extreme data scarcity, we constructed smaller training subsets. These subsets were generated by randomly selecting samples from each class in the training set, resulting in subsets named $D_{0.5}^{t}$ (23,141 images), $D_{0.2}^{t}$ (9,244 images), $D_{50}^{t}$ (1,850 images), $D_{30}^{t}$ (1,110 images), and $D_{20}^{t}$ (740 images), corresponding to 50%, 20%, 50 samples, 30 samples, and 20 samples from each class, respectively. Notably, we have the relationships $D_{20}^{t}\subseteq D_{30}^{t}\subseteq D_{50}^{t}$. Figure 3 visualizes the structure of these datasets.

### MAE algorithm

MAE is an SSL algorithm designed to improve feature representation by reconstructing input data from partial observations. Essentially, MAE introduces a masking strategy where a portion of the input (e.g., images) is randomly masked out, and the model is tasked with reconstructing these missing parts. The architecture of MAE typically comprises an encoder and a decoder. The encoder is responsible for processing the visible (unmasked) parts of the input, from which it derives a latent representation. This latent output is then used by the decoder to reconstruct the original input, with a specific focus on accurately predicting the masked regions. This approach forces the model to extract robust and meaningful feature representations. These representations can subsequently be fine-tuned leveraging the supervisory information for specific downstream tasks, such as the classification of diatoms.

### Experimental design

**Figure 4:**
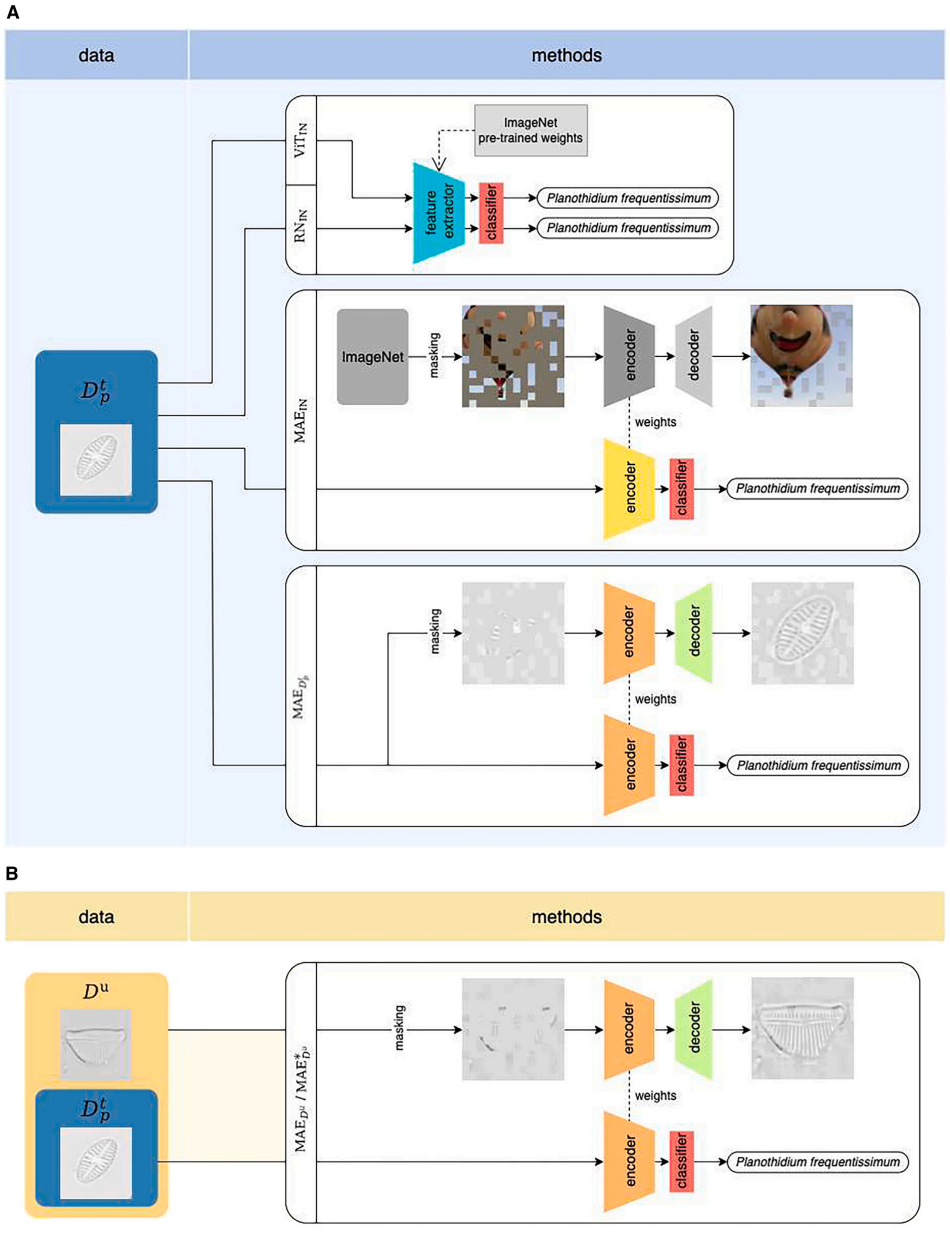
Overview of model pre-training and fine-tuning processes in this study (A) Utilization of exclusively labeled data (denoted as $D_{p}^{t}$, with *p* = 0.5, 0.2, 50, 30, 20 or none). This panel illustrates the fine-tuning process of ViT-L, ResNet-50 and MAE models on $D_{p}^{t}$, all initially pre-trained on ImageNet (denoted as $ViT_{IN}$, $RN_{IN}$, $MAE_{IN}$, respectively). Additionally, it depicts the fine-tuning of the $MAE_{D_{p}^{t}}$ model that has been pre-trained exclusively on the dataset $D_{p}^{t}$., (B) Inclusion of both labeled and unlabeled data (denoted as $D^{u}$). This panel describes the workflow wherein the MAE model, referred to as $MAE_{D^{u}}$/$MAE_{D^{u}}^{*}$ (pre-training phase: 800 epochs/1600 epochs), is initially pre-trained on the comprehensive dataset $D^{u}$. This dataset assembled by integrating all available annotated data, from which annotations have been removed, and additional unlabeled data. Following the pre-training phase, the model is fine-tuned on the annotated dataset $D_{p}^{t}$.

The experiments conducted in our study are depicted in Figure 4, which includes two sub-figures illustrating the different experimental setups under varying data availability scenarios. Figure 4A details the process employed when using exclusively given annotated data ($D_{p}^{t}$, with *p* = 0.5, 0.2, 50, 30, 20 or none). In this scenario, we fine-tuned ViT-L, ResNet-50, and MAE models initially pre-trained on ImageNet (referred to as $ViT_{IN}$., $RN_{IN}$, $MAE_{IN}$, respectively), with $D_{p}^{t}$, to analyze the effect of MAE and the impact of different volumes of data. Additionally, the MAE model was also solely pre-trained on $D_{p}^{t}$, referred to as $MAE_{D_{p}^{t}}$, and subsequently fine-tuned also with $D_{p}^{t}$.

Figure 4B illustrates the process of pre-training the MAE model on all available domain data, $D^{u}$, which includes all data regardless of annotation status, termed as $MAE_{D}^{u}$, followed by fine-tuning with $D_{p}^{t}$ (with *p* = 50, 30 or 20). This pre-training was further extended from 800 to 1600 epochs, with the model then referred to as $MAE_{D^{u}}^{*}$. In this scenario, we extract meaningful information from a substantial volume of domain data without annotations and fine-tune the information with a significantly reduced amount of labeled data, to investigate the effect of MAE in reducing the need for extensive annotations. Throughout our experiments, all parameters were kept consistent with the official MAE implementation settings, ensuring variations only in model architectures for our comparative analysis.

#### Evaluation metrics

Our primary metric for assessment is macro-average accuracy, as opposed to micro-average accuracy, due to the presence of class imbalance in our dataset. Micro-average accuracy aggregates the contributions of all instances to compute overall accuracy, which does not account for class size and can bias results toward classes with a higher number of instances. In contrast, macro-average accuracy computes the metric independently for each class before averaging them, thus each class equally contributes to the overall metric. This method is particularly advantageous in the classification of diatom data, where taxa disparity can skew performance metrics. By employing macro-average accuracy, we ensure a balanced evaluation, giving equal importance to the performance of smaller classes alongside larger ones, thereby providing a fairer and more representative measure of model performance. Additionally, we have assessed the macro-average F1-score to enhance the evaluation of classification performance comprehensively. To assess the robustness of our approach, we conducted experiments using three different seed settings chosen at random. This methodology guarantees that our findings are not dependent on a particular random initialization, thus offering a more reliable assessment of the model’s performance. Within the main text of our study, we present the mean macro-average accuracy across these three experimental seeds. Additionally, the macro-average accuracy for each individual seed is detailed in the Appendix.

### Optimal classification performance of our model

In the ‘Comparative MAE Performance’ experiments, our objective was to maximize the utilization of the given data to enhance classification performance. We fine-tuned $ViT_{IN}$, $RN_{IN}$, $MAE_{IN}$, $MAE_{D_{p}^{t}}$, and $MAE_{D}^{u}$ models using the full training set, $D^{t}$, to assess their performance across our dataset. Additionally, to explore the impact of dataset size on model performance, we fine-tuned the models on two smaller subsets, $D_{0.5}^{t}$ and $D_{0.2}^{t}$. All models were subsequently evaluated on the test set, $D^{\text{test}}$. The results of these experiments are summarized in Table 1. Here, we present the mean macro-average accuracy (expressed as a percentage) with comparisons to the baseline performance of the vision transformer initialized with ImageNet, $ViT_{IN}$. Detailed results for each seed are shown in Tables A1–A3 in Appendix A. The F1-scores are presented in Table B1 in Appendix B.

**Table 1:** Comparative MAE performance

Models	datasets ($D_{p}^{t}$)		
	$D^{t}$	$D_{0.5}^{t}$	$D_{0.2}^{t}$
$ViT_{IN}$	90.00	88.44	85.15
$RN_{IN}$	+0.97	+0.71	+0.81
$MAE_{IN}$	+1.99	$\underline{+2.27}$	$\underline{+2.91}$
$MAE_{D_{p}^{t}}$	$\underline{+2.36}$	+1.05	−3.86
$MAE_{D^{u}}$	+2.97	+3.52	+5.40

This table presents the mean macro-average accuracy of various models fine-tuned on the datasets $D^{t}$, $D_{0.5}^{t}$ and $D_{0.2}^{t}$ across three experimental seeds. The baseline performance (in percentage, %) is established by the vision transformer model initialized with ImageNet $ViT_{IN}$. Incremental improvements over the baseline (in percentage point, p.p.) are shown for the ResNet-50 initialized with ImageNet $\left(RN_{IN}\right)$ and the MAE initialized with ImageNet $\left(MAE_{IN}\right)$. Notably, the MAE model pre-trained on the given dataset $\left(MAE_{D_{p}^{t}}\right)$ demonstrates variable performance enhancements, with a significant decline noted in smaller dataset sizes. Highlighted values clearly reveal that our MAE model pre-trained on all available labeled and unlabeled data $\left(MAE_{D}^{u}\right)$ consistently outperforms other models. This superiority is particularly evident in smaller datasets.

The baseline $ViT_{IN}$ model achieved an accuracy of 90.00%, 88.44%, and 85.15% on $D^{t}$, $D_{0.5}^{t}$ and $D_{0.2}^{t}$, respectively. The ResNet-50 model initialized with ImageNet $\left(RN_{IN}\right)$ demonstrated slight improvements of approximately 1 p.p. over the baseline across all datasets. However, the MAE model initialized with ImageNet $\left(MAE_{IN}\right)$ exhibits more substantial enhancements, with performance increases of 1.99 p.p., 2.27 p.p., and 2.91 p.p. on $D^{t}$, $D_{0.5}^{t}$ and $D_{0.2}^{t}$, respectively. These improvements were more pronounced as the dataset size decreased. The MAE model pre-trained on the given labeled dataset $D_{p}^{t}$ $\left(MAE_{D_{p}^{t}}\right)$ recorded the highest improvement of 2.36 p.p. on $D^{t}$ but shows a significant decline in performance on the smallest dataset, $D_{0.2}^{t}$, indicating its sensitivity to dataset size. Remarkably, our MAE model pre-trained on the comprehensive dataset which includes all available labeled and unlabeled data, $MAE_{D}^{u}$, consistently outperformed all other models, particularly excelling on the smaller datasets $D_{0.5}^{t}$ and $D_{0.2}^{t}$, with improvements of 3.52 p.p. and 5.40 p.p., respectively.

### Enhanced efficiency in diatom classification through our model

In the ‘Annotation Effort Reduction Study’ experiments, to achieve high classification results using significantly reduced numbers of annotated data, we explore the potential of different models in this regard, with fine-tuning $ViT_{IN}$, $RN_{IN}$, $MAE_{IN}$, $MAE_{D}^{u}$, and $MAE_{D^{u}}^{*}$ models using $D^{t}$, $D_{50}^{t}$, $D_{30}^{t}$, and $D_{20}^{t}$ datasets, and evaluated the fine-tuned models on $D^{\text{test}}$. The results are presented in Table 2. Detailed results for each seed are shown in Tables A4–A6 in the Appendix A. The F1-scores are presented in Table B2 in Appendix B.

**Table 2:** Annotation effort reduction study

Models	datasets ($D_{p}^{t}$)			
	$D^{t}$	$D_{50}^{t}$	$D_{30}^{t}$	$D_{20}^{t}$
$ViT_{IN}$	90.00	81.06	77.42	73.59
$RN_{IN}$	–	82.02	78.73	74.95
$MAE_{IN}$	–	84.79	80.74	75.47
$MAE_{D^{u}}$	–	90.17	88.97	87.41
$MAE_{D^{u}}^{*}$	–	91.17	90.38	89.34

This table displays the mean macro-average accuracy (%) achieved by various models fine-tuned with very small datasets $D_{50}^{t}$, $D_{30}^{t}$ and $D_{20}^{t}$. Highlighted values indicate performances that, despite the significantly smaller dataset sizes, are notably comparable to the baseline established by the vision transformer initialized with ImageNet $\left(ViT_{IN}\right)$. This underscores the efficiency of the models in maintaining high accuracy with limited annotated data.

**Figure 5:**
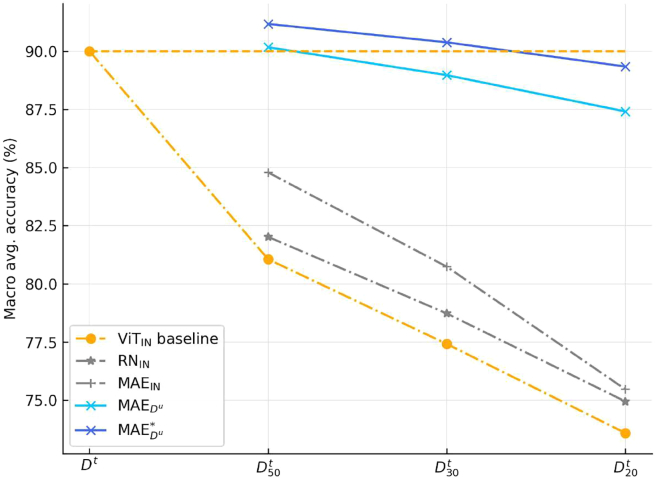
Our approach vs. baseline This figure graphically reveals the classification accuracy trends of different models fine-tuned on datasets of decreasing size, ranging from the full dataset to substantially reduced subsets ($D_{50}^{t}$, $D_{30}^{t}$, and $D_{20}^{t}$). The baseline performance is set by the vision transformer initialized with ImageNet $\left(ViT_{IN}\right)$, shown in orange. Notably, the $MAE_{D}^{u}$ model, when fine-tuned with $D_{50}^{t}$, and the $MAE_{D^{u}}^{*}$ model, when fine-tuned with $D_{30}^{t}$, achieve a classification accuracy comparable to the $ViT_{IN}$ baseline on the full dataset $D^{t}$.

The baseline performance was established by $ViT_{IN}$, which was fully supervised fine-tuned on the full training data, $D^{t}$, achieving an accuracy of 90.00%. The $RN_{IN}$ model consistently surpassed this baseline across all three subsets. Furthermore, the $MAE_{IN}$ model demonstrated superior performance compared to the $RN_{IN}$ model across the same subsets. As shown in the Figure 5, notable results were observed with the $MAE_{D}^{u}$ model, when fine-tuned with $D_{50}^{t}$, reached an accuracy of 90.17%, thereby exceeding the baseline performance on the full training set. Additionally, the longer pre-trained $MAE_{D^{u}}^{*}$ model, fine-tuned with $D_{30}^{t}$, achieved an accuracy of 90.38%.

## Discussion

The main goal of this work was to tackle the challenge of reducing the labeling efforts in DL-based diatom classification. We approached this problem from two perspectives: first, by enhancing classification performance of given annotated data; second, by minimizing the dependency on annotated data while maintaining high classification accuracy. This research filled the gap in the application of SSL for diatom classification, providing valuable insights into optimizing available resource utilization and minimizing the reliance on extensive annotation.

Previous studies have shown conflicting results regarding the classification performance of CNNs and ViTs. While some evidence suggests that CNNs outperform ViTs,^30^ other research points to the opposite.^24^ Owing to computational constraints and dataset variations, a comprehensive quantitative comparison to previously introduced approaches was not feasible in our study. Nevertheless, in our extensive real-world diatom image dataset, the $MAE_{IN}$ model consistently outperformed both $ViT_{IN}$ and $RN_{IN}$, particularly as dataset size decreased. Notably, the MAE model pre-trained on the given training set itself achieves the highest improvement on the full dataset $D^{t}$ but experienced performance declines on smaller subsets, likely due to limited SSL learning from smaller datasets. Consequently, when a given labeled dataset was sufficiently large, the domain dataset pre-trained $MAE_{D_{p}^{t}}$ exhibited optimal classification performance. Conversely, as the training set size decreased, the MAE model pre-trained with ImageNet $\left(MAE_{IN}\right)$ demonstrated superior results.

The integration of unlabeled data enabled the $MAE_{D}^{u}$ model, pre-trained on a comprehensive dataset encompassing all available data, to surpass all other models in classification accuracy, especially on smaller datasets. This demonstrates that the model has captured useful structural information from unlabeled data, thus underscoring its optimal utilization of available resources. Remarkably, although the dataset for pre-training is considerably smaller than ImageNet (1.2 million images), consisting of only 72,030 domain-specific images, its performance was outstanding. This achievement highlights the significant success of our approach in leveraging image-rich but label-poor domain-specific data for effective data utilization.

The reduction in the quantity of labeled data required for training presents substantial practical utility in life science applications, particularly where time and computational resources are limited. The success of our approach in diminishing the dependence on extensive annotation to achieve high classification performance is significant. In contrast to the fully supervised baseline set by the vision transformer initialized with ImageNet, $ViT_{IN}$, which necessitates fine-tuning on a full training set comprising 46,294 images, our model requires merely 50 annotated images per class across 37 classes. Furthermore, extending the pre-training duration from 800 to 1600 epochs has further decreased the necessary labeled data to 30 samples per class, thereby enhancing the cost-efficiency for domain experts. This extended training allows networks to learn more comprehensive knowledge, facilitating fine-tuning for specific downstream tasks. The finding is consistent with He et al.,^39^ indicating that a longer training schedule gives a noticeable improvement. This dramatic reduction is quantitatively significant as it enables the model to reach a comparable macro-average accuracy of approximately 90.0% with markedly fewer samples. The decrease in required annotation can be quantitatively expressed as follows:

$$\text{Percentage}\;\text{of}\;\text{annotation}\;\text{required}=\left(\frac{50\;\text{samples}/\text{class}\times 37\;\text{classes}}{46,294\;\text{samples}}\right)\times 100\approx 4.0\%\text{,}$$

$$\text{Percentage}\;\text{of}\;\text{annotation}\;\text{required}=\left(\frac{30\;\text{samples}/\text{class}\times 37\;\text{classes}}{46,294\;\text{samples}}\right)\times 100\approx 2.4\%\text{.}$$

**Figure 6:**
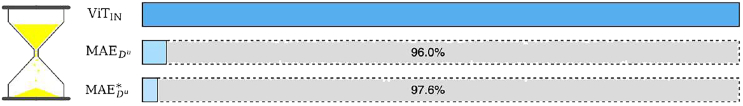
Annotation effort savings by using our self-supervised learning (SSL) model This figure illustrates the significant reduction in required annotation effort achieved by our $MAE_{D}^{u}$ and $MAE_{D^{u}}^{*}$ models in comparison to the $ViT_{IN}$ baseline. By fine-tuning the $MAE_{D}^{u}$ and $MAE_{D^{u}}^{*}$ models on the very small subsets $D_{50}^{t}$ and $D_{30}^{t}$, containing only 50 and 30 samples per class respectively, these models obtain classification accuracies comparable to the $ViT_{IN}$ model fine-tuned on the full dataset. This results in a reduction of required annotated data by approximately 96.0% and 97.6%, respectively, substantially conserving the efforts of domain taxonomic experts in annotation tasks.

As shown in Figure 6, the implementation of our $MAE_{D}^{u}$ and $MAE_{D^{u}}^{*}$ models significantly decreases the need for annotated data, achieving reductions in annotation effort by approximately 96.0% and 97.6%, respectively.

Overall, our results demonstrate that SSL can significantly alleviate the reliance on extensive annotation in diatom taxonomic classification. This is a valuable step for breaking through the research bottleneck of annotation scarcity in critical areas of life science such as water quality analysis, marine biology, and environmental studies. Our findings align with existing literature^40^ which highlights the benefits of SSL in scenarios with limited labeled data. By making our pre-trained models publicly available, we facilitate replication of our results, validation of our methodologies, and further exploration based on our findings. This initiative provides diatom researchers with a significant advantage in automatically analyzing their data, thereby making automatic classification more accessible and requiring considerably much less effort.

### Conclusions

In this study, we explore the potential of SSL to address a critical and enduring challenge in the DL-based classification of diatoms: the scarcity of annotated data. Our findings demonstrate that SSL pre-training yields significant benefits, particularly in scenarios where annotated data is scarce. The approach implemented and the pre-trained models provided publicly will significantly contribute to the field, enabling more efficient and accurate diatom taxonomy studies. By adopting this method, researchers are able to effectively utilize all available image data, thus substantially reducing reliance on traditional, labor-intensive manual labeling. This advancement is particularly beneficial for domain scientists tasked with analyzing large datasets.

### Limitations of the study

The main limitation of our method is the scarcity of annotated samples in many diatom classes, which restricted our study to only 37 classes, as shown in this paper. However, we expect that the volume of labeled diatom data will grow in the future and more classes can be added to the computational classification system.

## Resource availability

### Lead contact

Further information and requests for model implementation should be directed to and will be fulfilled by Tim W. Nattkemper (tim.nattkemper@uni-bielefeld.de).

### Materials availability

This study did not generate new unique reagents.

### Data and code availability

• Requests for the imaging data used in this study should be directed to the lead contact.
• The code associated with this work is available upon request from the lead contact.
• Any additional information necessary to reanalyze the data reported in this paper can be obtained from the lead contact upon request.

## Acknowledgments

This work was supported by the 10.13039/501100018929de.NBI Cloud within the 10.13039/501100018929German Network for Bioinformatics Infrastructure (de.NBI) and ELIXIR-DE (Forschungszentrum Jülich and W-de.NBI-001, W-de.NBI-004, W-de.NBI-008, W-de.NBI-010, W-de.NBI-013, W-de.NBI-014, W-de.NBI-016, W-de.NBI-022).

M.T., D.L., and M.K. were funded by the 10.13039/501100001659Deutsche Forschungsgemeinschaft (DFG, German Research Foundation; project number: 463395318, GZ: NA 731/11-1 & BE 4316/10-1).

## Author contributions

Conceptualization, M.T., D.L., and T.W.N.; methodology, M.T., D.L., and T.W.N.; formal analysis, M.T., D.L., and T.W.N.; investigation, M.T., D.L., and T.W.N.; recourse, M.T., D.L., M.K., and T.W.N.; data curation, M.T. and M.K.; writing – original draft, M.T.; writing–review and editing, M.T., D.L., M.K., and T.W.N.; supervision, D.L. and T.W.N.; project administration, T.W.N.; funding acquisition, T.W.N.

## Declaration of interests

The authors declare no competing interests.

## Declaration of generative AI and AI-assisted technologies in the writing process

During the preparation of this work the authors used ChatGPT in order to improve the readability and language of the manuscript. After using it, the authors reviewed and edited the content as needed and took full responsibility for the content of the published article.

## STAR★Methods

### Key resources table

**Table:** 

REAGENT or RESOURCE	SOURCE	IDENTIFIER
**Software and algorithms**		

ViT	Dosovitskiy et al.^28^	https://github.com/google-research/vision_transformer
MAE	He et al.^39^	https://github.com/facebookresearch/mae

**Others**		

de.NBI Cloud		https://www.denbi.de

### Method details

#### Evaluation metrics formulas

The formula for macro-average accuracy is given by:

$$\text{macro}-\text{average}\;\text{Accuracy}=\frac{1}{N}\sum_{i=1}^{N}\frac{TP_{i}+TN_{i}}{TP_{i}+TN_{i}+FP_{i}+FN_{i}}\text{,}$$

where N is the number of classes, $TP_{i}$, $TN_{i}$, $FP_{i}$, $FN_{i}$ are the number of true positives, true negatives, false positives, false negatives for class i, respectively.

The mean macro-average accuracy is defined as:

$$\text{mean}\;\text{macro}-\text{average}\;\text{Accuracy}=\frac{1}{S}\sum_{j=1}^{S}\left(\frac{1}{N}\sum_{i=1}^{N}\frac{TP_{ij}+TN_{ij}}{TP_{ij}+TN_{ij}+FP_{ij}+FN_{ij}}\right)\text{,}$$

where S is the number of different experimental seeds, N is the number of classes, and $TP_{ij}$, $TN_{ij}$, $FP_{ij}$, and $FN_{ij}$ represent the true positives, true negatives, false positives, and false negatives for class i in seed j, respectively.

#### Experimental settings

All experiments were conducted using images resized to 224×224 pixels. The models, including ViT-L, ResNet-50 and MAE, were implemented using the ‘timm’ library^42^ (version: 0.3.2). The entire framework was built on PyTorch^43^ (version: 2.0.0) and executed on an NVIDIA Tesla V100 PCIe with 16GB GPU.

##### Pre-training setting

The network is optimized using AdamW,^44^ with an optimizer momentum of $\beta_{1},\beta_{2}=0.9,0.95$.^45^ The weight decay is set to 0.05. The learning rate is scheduled using a cosine decay schedule,^46^ with a base learning rate of $1.5\times 10^{-4}$. The warmup epochs are 40. The batch size is set to 32. The augmentation strategy is RandomResizedCrop.

##### Fine-tuning setting

The network is optimized using AdamW, with an optimizer momentum of $\beta_{1},\beta_{2}=0.9,0.999$. The weight decay is set to 0.05. The learning rate is scheduled using a cosine decay schedule, with a base learning rate, layer-wise lr decay of $1\times 10^{-3}$, 0.75, respectively. The criterion is LabelSmoothingCrossEntropy, with a label smoothing set to 0.1. The model is trained for 100 epochs with warmup epochs set to 5. The weights are saved at the last epoch and loaded to evaluate performances on the test set. The batch size is set to 64. The augmentation strategy is RandAug (9, 0.5).^47^

### Quantification and statistical analysis

All analyses for calculating mean macro-average accuracy and F1 score were performed using Python (version 3.8) and scikit-learn (version 1.1.1).
