# Supplementary material for: Scaling down annotation needs: The capacity of self-supervised learning on diatom classification
Source: iScience. 2025 Mar 20;28(4):112236. doi: 10.1016/j.isci.2025.112236 (PMC12002600; doi:10.1016/j.isci.2025.112236)
Supplement: Document S1. Appendix A–C [file mmc1.pdf]

iScience, Volume *28*

Supplemental information

Scaling down annotation needs: The capacity of self-supervised learning on diatom classification

Mingkun Tan, Daniel Langenkämper, Michael Kloster, and Tim W. Nattkemper

Supplementary Information

Appendix A

Models	datasets (D^t_p)		
	D^t	$D^t_{0.5}$	$D^t_{0.2}$
ViT_{IN}	**90.00**	**88.61**	**84.99**
RN_{IN}	+0.97	+0.61	+1.41
MAE_{IN}	+1.99	<u>+2.20</u>	<u>+3.25</u>
$\text{MAE}_{D^t_p}$	<u>+2.36</u>	+0.90	-3.59
MAE_{D^u}	**+2.97**	**+3.61**	**+5.39**

Table A1. Comparative MAE Performance (seed = 10), Related to Table 1. This table presents the macro-average accuracy (%) of various models fine-tuned on the datasets D^t, $D^t_{0.5}$ and $D^t_{0.2}$, with improvements over the ViT_{IN} baseline (in percentage point, p.p.). Highlighted values clearly reveal that the MAE_{D^u} model consistently outperforms other models. This superiority is particularly evident in smaller datasets.

Models	datasets (D^t_p)		
	D^t	$D^t_{0.5}$	$D^t_{0.2}$
ViT_{IN}	**90.00**	**88.51**	**85.75**
RN_{IN}	+0.97	+0.40	+0.42
MAE_{IN}	+1.99	<u>+2.25</u>	<u>+2.76</u>
$\text{MAE}_{D^t_p}$	<u>+2.36</u>	+0.91	-4.33
MAE_{D^u}	**+2.97**	**+3.38**	**+5.25**

Table A2. Comparative MAE Performance (seed = 100), Related to Table 1. This table presents the macro-average accuracy (%) of various models fine-tuned on the datasets D^t, $D^t_{0.5}$ and $D^t_{0.2}$, with improvements over the ViT_{IN} baseline (in percentage point, p.p.). Highlighted values clearly reveal that the MAE_{D^u} model consistently outperforms other models. This superiority is particularly evident in smaller datasets.

Models	datasets (D^t_p)		
	D^t	$D^t_{0.5}$	$D^t_{0.2}$
ViT_{IN}	**90.00**	**88.19**	**84.71**
RN_{IN}	+0.97	+1.12	+0.59
MAE_{IN}	+1.99	<u>+2.36</u>	<u>+2.71</u>
$\text{MAE}_{D^t_p}$	<u>+2.36</u>	+1.34	-3.67
MAE_{D^u}	**+2.97**	**+3.57**	**+5.55**

Table A3. Comparative MAE Performance (seed = 1000), Related to Table 1. This table presents the macro-average accuracy (%) of various models fine-tuned on the datasets D^t, $D^t_{0.5}$ and $D^t_{0.2}$, with improvements over the ViT_{IN} baseline (in percentage point, p.p.). Highlighted values clearly reveal that the MAE_{D^u} model consistently outperforms other models. This superiority is particularly evident in smaller datasets.

Models	datasets (D^t_p)			
	D^t	D^t_{50}	D^t_{30}	D^t_{20}
ViT_{IN}	**90.00**	**80.85**	**76.86**	**72.76**
RN_{IN}	-	82.27	78.97	74.69
MAE_{IN}	-	84.99	80.64	75.19
MAE_{D^u}	-	**90.03**	88.67	86.30
$\text{MAE}^*_{D^u}$	-	90.89	**90.33**	88.49

Table A4. Annotation Effort Reduction Study (seed = 10), Related to Table 2. This table displays the macro-average accuracy (%) achieved by various models fine-tuned with very small datasets D^t_{50}, D^t_{30} and D^t_{20}. Highlighted values indicate performances that are notably comparable to the ViT_{IN} baseline, despite the significantly smaller dataset sizes.

Models	datasets (D^t_p)			
	D^t	D^t_{50}	D^t_{30}	D^t_{20}
ViT_{IN}	**90.00**	**81.49**	**77.68**	**73.72**
RN_{IN}	-	81.92	78.93	75.79
MAE_{IN}	-	84.61	80.41	75.36
MAE_{D^u}	-	90.39	89.44	88.42
$\text{MAE}^*_{D^u}$	-	91.30	90.52	89.48

Table A5. Annotation Effort Reduction Study (seed = 100), Related to Table 2. This table displays the macro-average accuracy (%) achieved by various models fine-tuned with very small datasets D^t_{50}, D^t_{30} and D^t_{20}. Highlighted values indicate performances that are notably comparable to the ViT_{IN} baseline, despite the significantly smaller dataset sizes.

Models	datasets (D^t_p)			
	D^t	D^t_{50}	D^t_{30}	D^t_{20}
ViT_{IN}	**90.00**	**80.82**	**77.71**	**74.30**
RN_{IN}	-	82.59	78.30	74.38
MAE_{IN}	-	84.77	81.18	75.85
MAE_{D^u}	-	90.08	88.81	87.52
$\text{MAE}^*_{D^u}$	-	91.33	90.29	90.05

Table A6. Annotation Effort Reduction Study (seed = 1000), Related to Table 2. This table displays the macro-average accuracy (%) achieved by various models fine-tuned with very small datasets D^t_{50}, D^t_{30} and D^t_{20}. Highlighted values indicate performances that are notably comparable to the ViT_{IN} baseline, despite the significantly smaller dataset sizes.

Appendix B

Models	datasets (D_p^t)		
	D^t	$D^t_{0.5}$	$D^t_{0.2}$
ViT_{IN}	**90.54**	**89.09**	**86.15**
RN_{IN}	+0.71	+0.40	+0.71
MAE_{IN}	+1.73	<u>+1.94</u>	<u>+2.52</u>
$\text{MAE}_{D_p^t}$	<u>+1.84</u>	+0.96	-3.52
MAE_{D^u}	**+2.41**	**+3.02**	**+4.77**

Table B1. Comparative MAE Performance (F1-score), Related to Table 1. This table presents the macro-average F1-score (%) of various models fine-tuned on the datasets D^t, $D^t_{0.5}$ and $D^t_{0.2}$, with improvements over the ViT_{IN} baseline (in percentage point, p.p.). Highlighted values clearly reveal that the MAE_{D^u} model consistently outperforms other models. This superiority is particularly evident in smaller datasets.

Models	datasets (D_p^t)			
	D^t	D^t_{50}	D^t_{30}	D^t_{20}
ViT_{IN}	**90.54**	**71.21**	**66.17**	**61.99**
RN_{IN}	+0.71	+1.00	+2.06	+1.11
MAE_{IN}	+1.73	+5.83	+5.90	+3.62
MAE_{D^u}	**+2.41**	**+14.70**	**+18.38**	**+20.51**
$\text{MAE}^*_{D^u}$	**+2.50**	**+15.79**	**+20.16**	**+22.51**

Table B2. Annotation Effort Reduction Study (F1-score), Related to Table 2. This table displays the macro-average F1-score (%) achieved by various models fine-tuned with very small datasets D^t_{50}, D^t_{30} and D^t_{20}, with improvements over the ViT_{IN} baseline (in percentage point, p.p.). Highlighted values clearly reveal that the MAE_{D^u} and $\text{MAE}^*_{D^u}$ models consistently outperforms other models. This superiority is particularly evident in smaller datasets.

Appendix C

We applied our pre-trained models MAE_{D^u} and $\text{MAE}^*_{D^u}$ to two additional diatom image datasets, Aqualitas[48] and Dorel[49], to evaluate their applicability and effectiveness within the diatom research community, especially in cases where no sufficient unlabeled data are available. The Aqualitas dataset consists of 10,472 images, which encompass 100 distinct diatom taxa. We allocated 80% of these images to a primary training set, designated as A^t, and the remaining 20% to a test set, denoted as A^{test}. The Dorel dataset contains 9,772 images across 166 distinct taxa. Due to the minimal class size of 20 images in this dataset, which is insufficient for reliable evaluation, we included classes containing at least 50 images for our experiments. This selection process resulted in 94 distinct classes totaling 7,380 images, which were similarly divided into training and test sets, named E^t and E^{test}, respectively. To investigate scenarios of data scarcity, we created smaller training subsets, A^t_{30}, E^t_{40}, and E^t_{30}, containing 30, 40, and 30 samples from each class, respectively (The minimal class size are 32 and 40 images in A^t and E^t.). Notably, $E^t_{30} \subseteq E^t_{40}$.

We fine-tuned ViT_{IN}, RN_{IN}, MAE_{IN}, MAE_{D^u}, and $\text{MAE}^*_{D^u}$ models using A^t and A^t_{30}, E^t, E^t_{40}, and E^t_{30} datasets, and evaluated the fine-tuned models on A^{test}, and E^{test} respectively. The results of these experiments are summarized in Table C1 and Table C2, respectively.

Models	Accuracy		F1-score	
	A^t	A^t_{30}	A^t	A^t_{30}
RN_{IN}	**97.38**	**90.34**	**97.41**	**93.16**
ViT_{IN}	+0.48	+1.43	+0.45	+1.18
MAE_{IN}	+0.87	+2.57	+0.87	+2.49
MAE_{D^u}	+0.87	+2.40	+0.83	+2.42
$\text{MAE}^*_{D^u}$	+0.94	+2.73	+0.88	+2.82

Table C1. Performance of our models on the Aqualitas dataset. This table presents the macro-average accuracy (%) and macro-average F1-score (%) of various models fine-tuned on the datasets A^t and A^t_{30}, with improvements over the RN_{IN} baseline. Highlighted values clearly reveal that our $\text{MAE}^*_{D^u}$ model consistently outperforms other models. This superiority is particularly evident in smaller datasets, aligning with the results presented in the main text.

Models	Accuracy			F1-score		
	E^t	E^t_{40}	E^t_{30}	E^t	E^t_{40}	E^t_{30}
RN_{IN}	**95.70**	**93.47**	**91.19**	**95.65**	**93.13**	**90.82**
ViT_{IN}	+0.34	+0.25	+0.86	+0.40	+0.10	+0.82
MAE_{IN}	+1.11	+0.92	+1.81	+1.15	+0.86	+1.72
MAE_{D^u}	+0.95	+0.75	+1.36	+1.13	+0.65	+1.42
$\text{MAE}^*_{D^u}$	**+1.21**	**+1.65**	**+2.46**	**+1.30**	**+1.78**	**+2.51**

Table C2. Performance of our models on the Dorel dataset. This table presents the macro-average accuracy (%) and macro-average F1-score (%) of various models fine-tuned on the datasets E^t, E^t_{40} and E^t_{30}, with improvements over the RN_{IN} baseline. Highlighted values clearly reveal that our $\text{MAE}^*_{D^u}$ model consistently outperforms other models. This superiority is particularly evident in smaller datasets, aligning with the results presented in the main text.
